# Evaluating a Campus Food Pantry’s Potential Impact on Nutrition Security using the Reach, Effectiveness, Adoption, Implementation, and Maintenance (RE-AIM) Framework

**DOI:** 10.1016/j.cdnut.2023.101984

**Published:** 2023-08-01

**Authors:** Ana I. Mitchell, Melissa P. Prescott

**Affiliations:** 1 Division of Nutritional Sciences, University of Illinois at Urbana, Champaign, IL, United States; 2 Division of Nutritional Sciences and Food Science and Human Nutrition, University of Illinois at Urbana, Champaign, IL, United States

**Keywords:** nutrition security, food pantry, university, food insecurity, college students, implementation science, charitable food system

## Abstract

**Background:**

Campus food pantries are uniquely positioned to promote health equity with the foods they make accessible to food-insecure students.

**Objectives:**

This study assessed the implementation and effectiveness of a client-choice campus food pantry to understand its potential impact on nutrition security and to inform future implementation.

**Methods:**

This observational study was designed using the reach, effectiveness, adoption, implementation, and maintenance framework, leveraging administrative data collected from a client-choice campus food pantry at a large Midwest university from August 2020 to May 2022. Pantry staff tracked student usage and item inventory. Items were analyzed for specific nutrients using the Nutrition Data System for Research. The mean nutrients and servings per food group distributed per visit were calculated and compared with dietary recommendations (effectiveness). Characteristics of pantry operation and setup were detailed (adoption). The percentage of openings with adequate stock to meet distribution guidelines was determined (implementation). Descriptive statistics were calculated, and multiple linear regressions determined whether significant changes in nutrients and food were distributed based on distribution guidelines and stock available.

**Results:**

Effectiveness: Vitamin D, fruits, vegetables, and whole grains were below 3 d of the recommended intake for all students, and energy, fiber, potassium, vitamin A, and grains were low for male students. Adoption: The pantry was established through a collaboration with a pre-existing community food pantry and operates as a 501(c) and is managed by campus recreation staff. Implementation: The pantry supplied enough produce for 72% of openings to meet distribution guidelines and enough dairy for 63% of openings.

**Conclusions:**

The food and nutrients distributed in limited amounts are consistent with those underconsumed according to the Dietary Guidelines for Americans. More research is needed to understand how pantry implementation can best support nutrition security through the adequate distribution of key nutrients and food groups.

## Introduction

Food insecurity is a barrier to higher educational attainment [1] and having a college degree is associated with numerous health and social advantages across the lifespan [[2], [3], [4], [5]]. Up to 40% of college students face food insecurity [6] and the students at greatest risk are often underrepresented in higher education. Establishing campus food pantries is the primary way universities have sought to support food-insecure students [7]. Traditionally, food assistance programs like food pantries were designed to improve food security, focusing largely on food sufficiency and adequate calories to avoid hunger [8]. In an effort to promote health equity given burgeoning health disparities and lower dietary quality among food-insecure individuals [9], the focus has shifted to improving nutrition security. This is defined as providing consistent access, availability, and affordability of foods and beverages that promote well-being and prevent disease [10,11]. Campus food pantries are therefore uniquely positioned to promote health equity with the foods they make accessible to students, especially for the most vulnerable who rely on food pantries chronically [12] or as their sole source of food [13].

College students tend to consume nutrient-poor diets [14,15], and food-insecure college students are at even higher risk of inadequate nutrition [16]. A systematic review examining the association between food insecurity and dietary outcomes among university students found that food-insecure students consumed lower amounts of healthy foods (for example, fruits, vegetables, and whole grains) and more unhealthy foods (for example, fast foods, added sugars, and sugar-sweetened beverages) than their food-secure counterparts [16]. Among adults, another systematic review found that food-insecure individuals tend to underconsume energy, fruits, vegetables, dairy products, and calcium. They are at risk for deficiencies in vitamins A, C, D, and B, iron, magnesium, and zinc [17]. These disparities in dietary quality make nutrition security a critical focus for food assistance programs in higher education.

There is limited research investigating the nutritional quality of food distributed from both campus food pantries and client-choice pantries; however, food delivered from traditional pantries is well-documented [18]. Client-choice food pantries allow individuals to select their food while traditional food pantries provide prepackaged bags of food [19]. Client-choice pantries honor food preferences and dietary restrictions by allowing clients to choose the food they can eat, know how to prepare, and select items they do not already have [20,21]. Research from traditional pantries found nutritional deficiencies in foods offered, such as limited amounts of vitamins A and C, calcium, and dairy products [18]. More research is needed to determine the food received and associated nutrients from client-choice pantries.

Among campus food pantries, only 2 studies describe the nutritional value of foods provided. One study investigated the nutritional quality and amount of food received by students from 3 food pantries accessible to college students in rural North Carolina [22]. In this study, only 1 pantry was on-campus and only 1 was a client-choice pantry. Based on the dietary requirements of an active 20-y-old male for 14 d, they found that the 3 pantries combined provided 38% of total daily calories. It also showed below recommended daily levels for vitamin C (27%) and D (5%), potassium (29%), calcium (38%), and above levels for sugar (220%) and trans-fat (342%) [22]. Another study conducted at a small southeastern university used data visualization software to study the food distributed from a campus food pantry [23]. They found that students preferred essential foods like pasta and canned vegetables over snack foods like Pop-Tarts and cookies [23]. No other studies have identified the nutrients and food received from a client-choice campus food pantry and its contribution to dietary recommendations.

The AHA recommends assessing whether the food provided by the charitable food system has nutritional quality consistent with the United States Dietary Guidelines for Americans (DGA) [8]. However, providing enough healthy food may only be part of the solution. A recent study found that the nutritional quality of foods selected at a food pantry was not associated with the nutritional quality of the food stocked [24]. Therefore, pantry implementation (for example, the use of distribution guidelines, adequate stock, and the pantry setup) may play a role in the nutritional quality of foods received. To assess a campus food pantry’s ability to promote nutrition security, the Reach, Effectiveness, Adoption, Implementation, and Maintenance (RE-AIM) framework was used to assess both the implementation and impact of a program operating under real-world conditions and constraints [25]. The RE-AIM evaluation framework provides information about how and to what extent a program works in the real-world environment, including how a program is being used, whether it is implemented as intended, and having the expected impact [26]. During the first year of operation, ∼20% of students were aware of the campus food pantry and the program reached 3.1% of food-insecure students [12]. This study aimed to assess program effectiveness (the amount of key nutrients and servings per food group delivered to students), adoption (characteristics of the organization and how the program was set up), and implementation (consistency of program delivery, and adaptations). Maintenance was not assessed in the present study.

## Methods

### Study design and participants

The present study used secondary and administrative data collected from a satellite campus food pantry (the Food Assistance & Well-being Program) during the first 2 y of operation (August 2020 to May 2021, and August 2021 to May 2022) at a large Midwestern public university. The program goal was to provide acute supplemental food assistance to students in need and ensure access to nutritious food. Students could visit as needed without restriction. To use the pantry, students had to create a profile with an online program, Link2Feed. The Link2Feed system has been explained elsewhere [12] and provides user demographic data. The study protocol was deemed exempt by the Institutional Review Board at the University of Illinois at Urbana-Champaign (IRB #21148) before the pantry opening.

### Food pantry characteristics and measures

#### Food pantry characteristics

The campus food pantry was open twice a week (Tuesday 1–4 pm and Saturday 2–5 pm) at the campus recreation center. During the first year, the pantry implemented distribution guidelines based on MyPlate: students could take up to 4 fruits, 4 vegetables, 2 grains, 2 proteins, 4 dairy items or dairy substitutes, and 4 snacks or personal items for up to 20 items total. During the second year, no distribution guidelines were implemented until November, when the number of items students could take per food category was limited. Limits were set at 5 produce items, 3 dairy items, or refrigerated items, 2 grains, 3 breakfast items, 1 frozen meat, 2 freezer items, 2 canned items, 2 snacks, and 2 miscellaneous items for a total of 22 items. During both years, guidelines on what could be selected from the pantry were created by graduate dietetics students that oversaw pantry operations and were implemented using shelf tags. The pantry received food from the parent food pantry, food bank, donations, and other campus programs serving as a food rescue program.

#### Program reach

Most student users during the first 2 y of operation (August 2020 to May 2022) were female (59.9%), Asian/Pacific Islander (35.9%), employed (62.8%), and 22 y or older (53.2%). Approximately half of the students visited once and most students relied on the pantry for 1 mo (62.1%). Approximately 15% of students, visited 8 or more times during an academic year and these students had a longer span of use (an average of 6.5 mo between their first and last visits), visited more frequently (on average 2 wk between visits), and were more likely to be graduate students and older [12].

#### Pantry inventory

Pantry staff and volunteers maintained a pantry inventory spreadsheet in Excel. The inventory included the name, brand, and net weight of each item and the amount available at the start and end of each pantry opening. Unpackaged produce was weighed on a gram scale. From August 2020 to May 2021, a research assistant photographed each item during restocking, including the front of the package label and the nutrition facts panel and ingredients.

Once the inventory was shared with researchers, research assistants assigned each item a unique ID using a systematic naming procedure and labeled the respective photographs with the same ID. Pantry items were entered into the Nutrition Data System for Research (NDSR) version 2020 and 2021 by trained research assistants using the photos for reference. Standardized recipes were entered for items that were donated from campus programs. Exact items not found in NDSR (for example, brand or flavor) used a generic version or substitute with a similar nutrient profile. If an acceptable substitute could not be found, an item was created as a recipe using individual ingredients or submitted as a new food item to NDSR. All pantry items were coded in NDSR with their item ID. All entries were assessed for accuracy by a second research assistant.

### Statistical analysis

The pantry inventories and NDSR Food File (File 02), User-recipe Properties Totals File (File 05), and NCC Food Group Serving Count System Output File Specifications Serving Count Food File (File 07) were merged using item ID. Each item was coded for food group analysis using NCC Food Group Serving Count System Subgroups using Additional Files outlined in Appendix 10 of the NDSR User Manual. Before analysis, missing data were addressed. Three pantry openings (2.4% of the total data) were dropped from the analyses given inventory data was not logged on 3 occasions. There were 6 pantry openings missing demographic data that were imputed using a carry backward method [27], given characteristics of pantry users were relatively stable within a given week. This method was more sensitive than using mean imputation, given trends in student usage varied throughout the year [12].

Descriptive statistics were calculated to determine the number of nutrients and servings per food group received on average per visit per year. Distribution amounts were calculated based on the amount of food distributed per pantry opening and the number of visitors, rather than based on what each student selected. Nutrients were compared with Dietary Reference Intakes (DRI) for both males and females aged 19–30 y. Servings per food group were compared with the DGA using the 2200 and 2800 calorie-level patterns, for females and males, respectively. These calorie levels correspond with the energy requirements for 19–26-y-olds with moderate activity levels. Moderate activity is needed to promote the prevention of chronic disease [28] and has been used in related studies [18,22]. Levels of added sugar and saturated fat were determined using the DGA (that is, less than 10% of daily calories) and the calorie-level patterns for male and female students. The macronutrient distribution of the total calories for protein, carbs, and fat and the percentage of calories from added sugar was calculated and compared with Acceptable Macronutrient Distribution Ranges (AMDR) and the DGA guidance on added sugar. Linear regressions assessed the difference in items and grams of food distributed based on distribution guidelines (year 1 MyPlate guidelines compared with year 2 Limits), and based on adequate stock for dairy and produce. Models were adjusted for pantry stock (number of items or grams per food available per category and per visitor), the number of visitors, visitor demographics (race as percentage of White, gender as percentage of female, and age), and month fixed effects. The capacity of the pantry to meet distribution guidelines based on the number of pantry visitors was graphed for dairy, grain, and produce. The characteristics of the organization and implementation site including the setting, staff, setup, and operation over 2 y were described. All analysis was conducted in STATA/MP 14.1 (StataCorp, LP).

## Results

### Effectiveness

**FIGURE 1:**
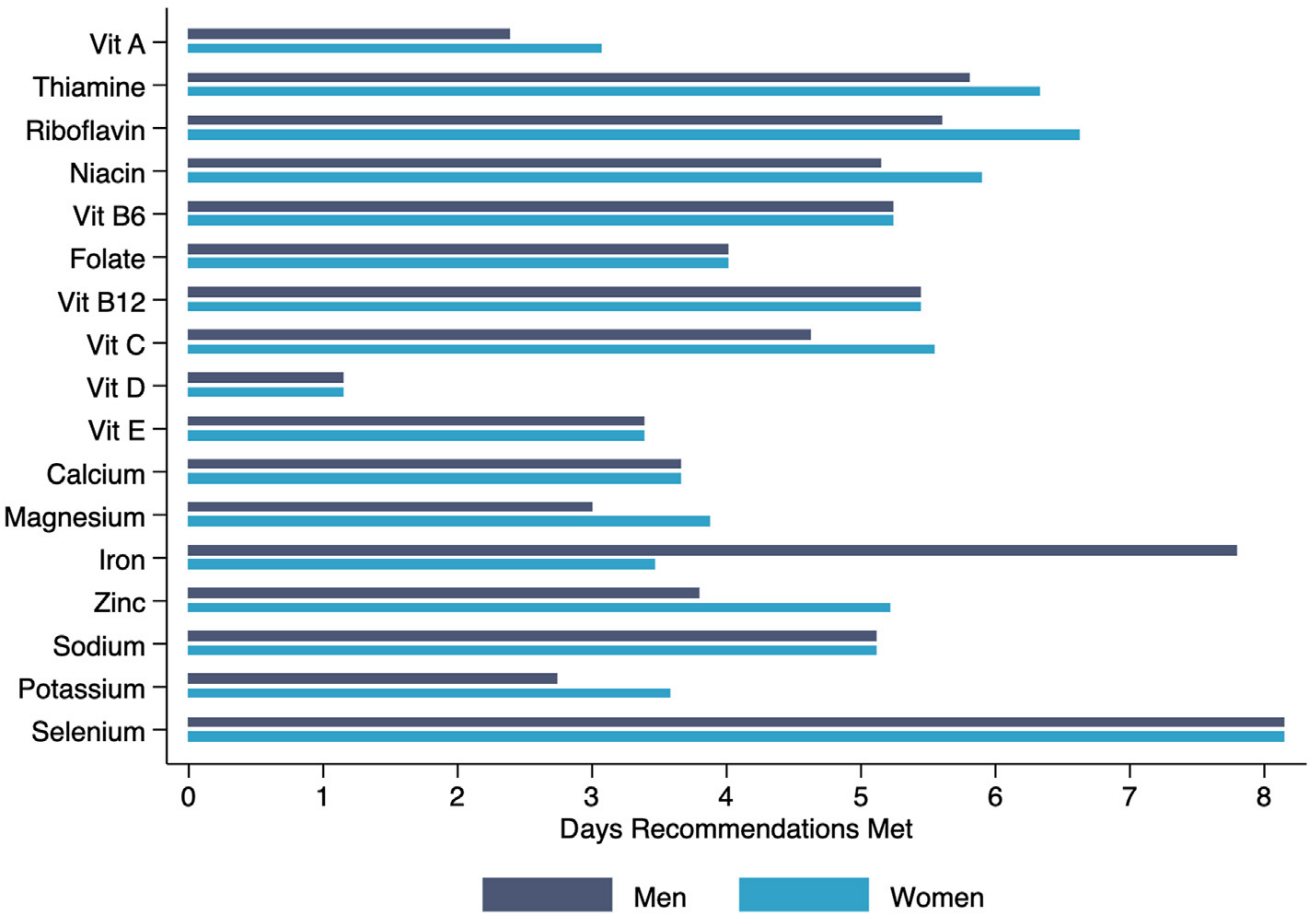
The number of days Dietary Reference Intakes were met for male and female students aged 19–30 y based on the average amount of micronutrients received per food pantry visit from August 2020 to May 2022.

**FIGURE 2:**
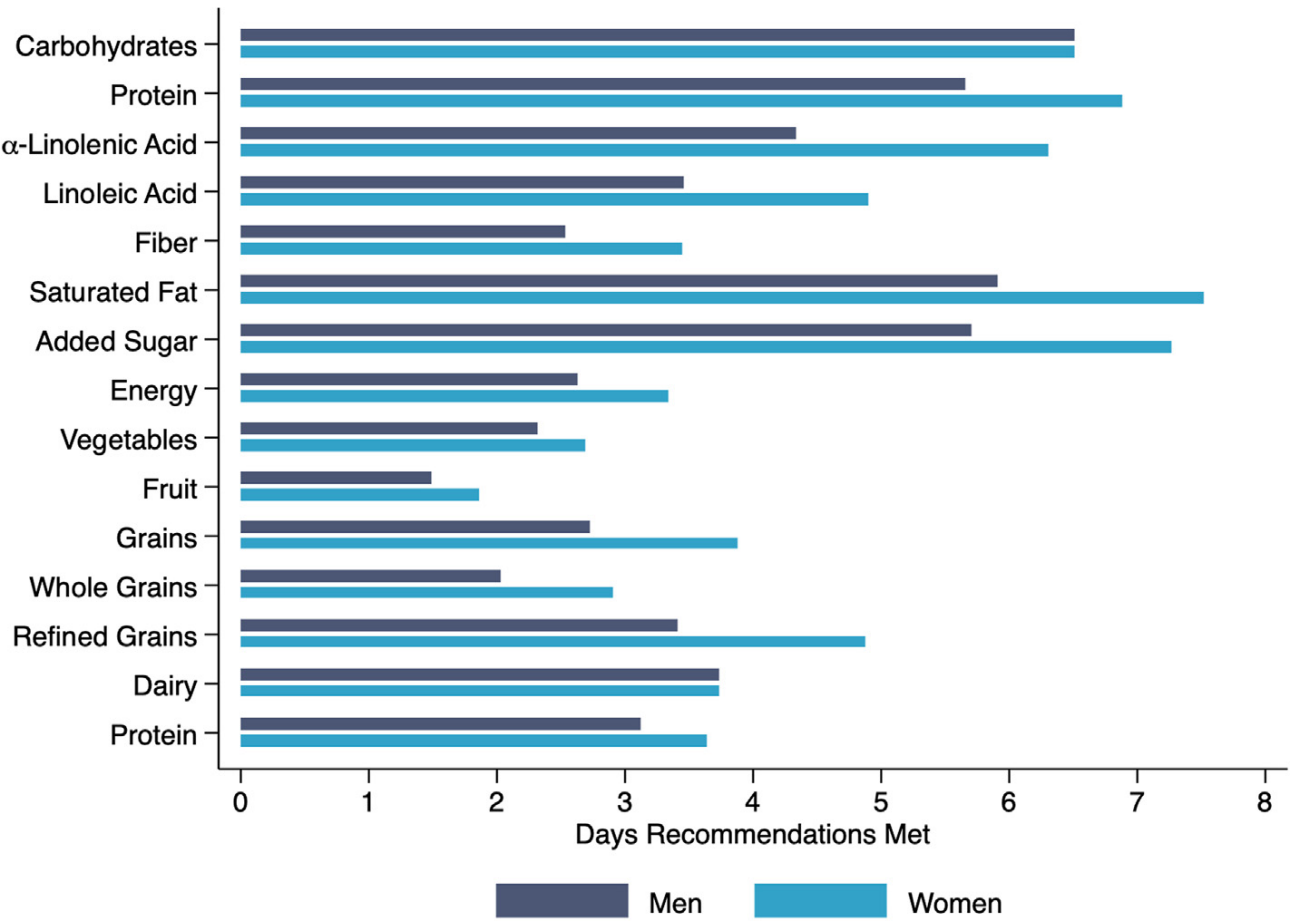
The number of days Dietary Reference Intakes and recommendations from the Dietary Guidelines for Americans were met for male and female students aged 19–30 based on the average amount of macronutrients and servings per food group received per food pantry visit from August 2020 to May 2022. Calorie-level patterns correspond to the estimated needs of 19–26-y olds with moderate activity levels for females (2200 kcal) and males (2800 kcal).

FIGURE 1, FIGURE 2 present the number of days DRI or DGA are met based on the average amount of micronutrients, macronutrients, or servings per food group received per visit. On average, females receive more days of adequate nutrients than male students, given DRI requirements for females are lower than males, except for iron. Most nutrients were supplied to fulfill at least 3 d of recommended intake except for vitamin D, fruits, vegetables, and whole grains for both males and females. Energy, fiber, potassium, vitamin A, and grains were also under the 3-d recommended intake for males. Sodium, saturated fat, and added sugar were above 5 d of recommended intake with energy meeting only ∼3 d for males and females.

The change in the number of items and amount of food distributed using different distribution guidelines (year 1 compared with year 2) is presented in Table 1. The distribution guidelines implemented in year 2 during 41 pantry openings provided, on average, 2 more items of dairy, vegetables, and items of food categorized as “other” (that is, mixed dishes, snacks, condiments, cooking staples, beverages, or desserts), and an additional item of fruit and grains. When comparing the amount of food distributed in grams or milliliters, there were more protein, grain, and other items distributed.

**TABLE 1:** The number of items and the amount of food distributed by distribution guidelines implemented in year 1 (*n* = 62 openings) and year 2 (*n* = 41 openings) with food grouped by category

	Change in number of items distributed *β* (95% CI)	*P*	Grams or milliliters distributed *β* (95% CI)	*P*
Dairy	2.37 (1.34, 3.41)	0.000	338.47 (−49.39, 726.33)	0.086
Protein	0.78 (−0.18, 1.76)	0.110	387.46 (99.63, 675.28)	0.009
Produce	1.47 (−0.17, 3.11)	0.078	468.95 (−200.24, 1138.14)	0.167
Fruit	1.32 (0.39, 2.26)	0.006	−147.26 (−495.37, 200.85)	0.402
Vegetables	1.96 (0.61, 3.32)	0.005	621.63 (−47.58, 1290.83)	0.068
Grain	1.34 (0.14, 2.54)	0.029	387.25 (183.63, 590.87)	0.000
Other	2.56 (1.00, 4.11)	0.002	710.87 (413.51, 1008.24)	0.000

Linear regression models were adjusted for stock (number of items or grams per food available per category and per visitor), total visits per opening, visitor demographic characteristics (age, race/ethnicity, gender), and month fixed effects.

The AMDR ranges for the percentage of calories from carbohydrates, fat, and protein are as follows. On average, 48.20% of calories came from carbohydrates (52.29% in year 1 and 44.04% in year 2), 38.09% of calories came from fat (34.89% in year 1 and 41.34% in year 2), and 16.75% came from protein (16.35% in year 1 and 17.16% in year 2). The percentage of calories from added sugar was under 10% at an average of 8.92% (9.22% in year 1 and 8.61% in year 2).

### Adoption

The campus pantry was developed as a satellite of a pre-existing community food pantry and operates as a 501(c). The program is housed within the campus recreation center and operates as a Student Wellness Program, overseen by the Associate Director of Assessment and Student Wellness at the Campus Recreation Center. Two graduate students hold assistantships with the recreation center and manage pantry operations each academic year, including restocking, training, and coordination of undergraduate volunteers and staff. The graduate students had a background in dietetics and oversaw food orders from the foodbank and supplementary food purchased from Walmart. They also determined operational procedures (that is, distribution guidelines) and were present at some pantry openings. At each pantry opening, there was 1 paid staff member (an undergraduate student) employed by the recreation center who supervised 3–4 pantry volunteers that helped set up and log the inventory. The setup included moving mobile shelving units and produce bins out of a storage closet to set them up within the instructional kitchen. The storage closet was attached to the instructional kitchen and housed 2 refrigerators and 2 freezers used to store pantry items.

Initially, the pantry received food primarily from the parent food pantry that received shipments from the foodbank and donations; this took place once a week on Mondays. As the pantry grew during year 1, the pantry started to restock again on Fridays, as needed, and later began restocking food directly from the foodbank. The pantry also received donations and food from a campus café, the sports program, and university dining, serving as a food rescue program. Food from the campus café was primarily salads, fruit cups, vegetable cups with ranch, and desserts. The sports program only donated food during the first year of operation; food included sandwiches, wraps, oatmeal cups, salads, and fruit cups. The recreation center also hosted food drives where bringing a certain number of nonperishable food items to the pantry allowed them to join workout classes for free. Supplemental food purchased for the pantry supported campus programs like the sustainable farm and meat science laboratory, and other items were purchased from Walmart.

### Implementation

**FIGURE 3:**
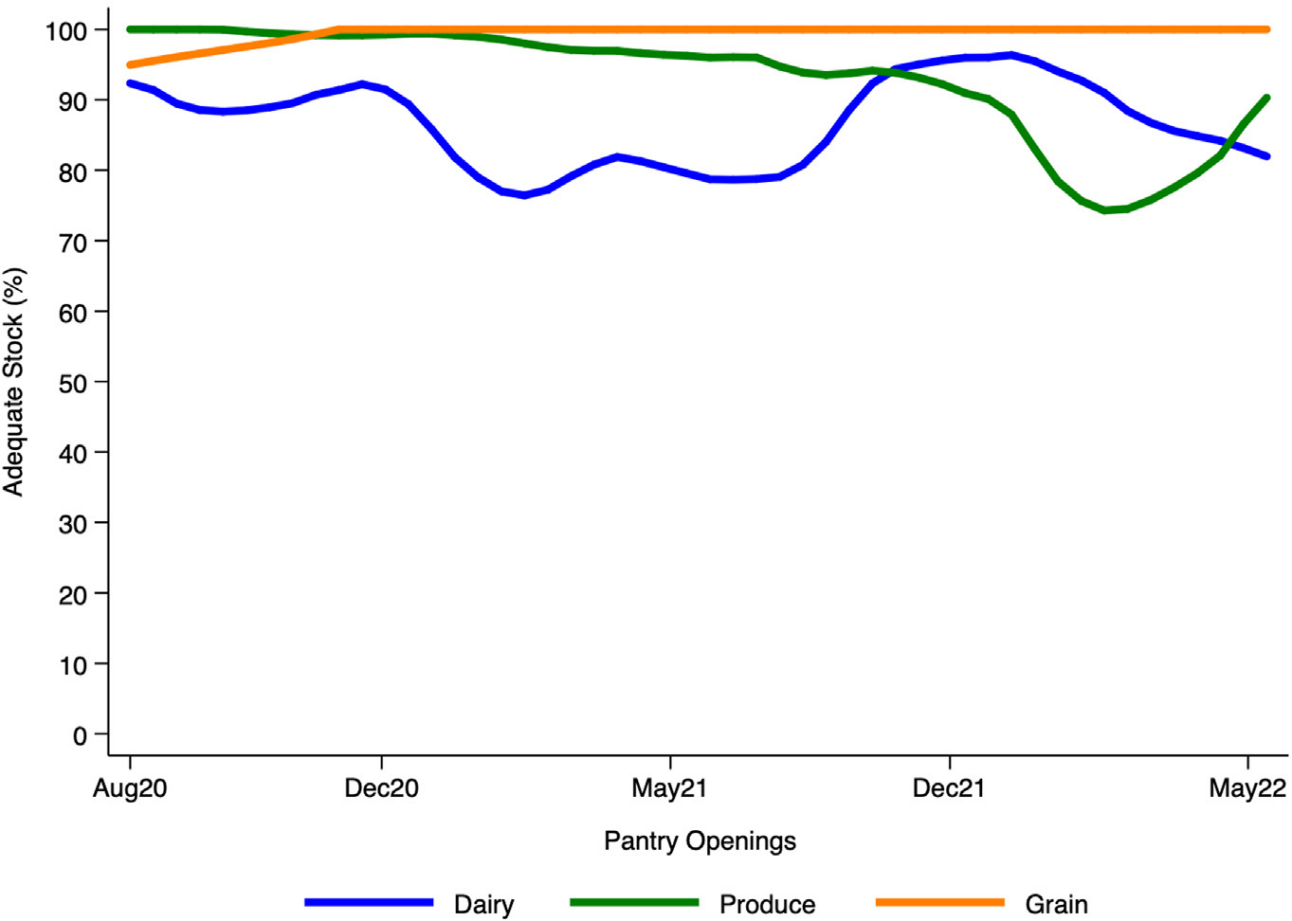
The capacity to meet distribution requirements based on the number of visitors per pantry opening was graphed as the percentage of adequate stock from August 2020 to May 2022.

Adequate stock for key food groups was assessed to determine the consistency of program delivery. The pantry did not have adequate stock to meet distribution guidelines in year 2 for 11 out of 41 pantry openings. The openings without adequate stock had an average number of 34 visitors compared with other openings that served an average of 24 students. The pantry operated on a “first come, first served” basis. On low-inventory days, implementation did not change (for example, students were not made aware of low stock or told to grab additional items from other food categories if some food categories ran out of stock). The percentage of adequate stock required to meet distribution guidelines based on the number of visitors per pantry opening for dairy, produce, and grains are graphed in Figure 3.

There was not enough dairy to meet guidelines for 33.98% of pantry openings (40.32% during year 1 and 24.39% during year 2). Produce stocked was insufficient for 27.18% of pantry openings (14.52% during year 1 and 46.34% during year 2) and grains were sufficiently stocked for all openings except for 1 during year 1. Figure 3 shows adequate stock for dairy fluctuates and was higher in year 2 while having enough produce stocked decreased from year 1 to year 2.

Another component related to whether the pantry was implemented as intended is whether guidelines were followed. The average items distributed per food category is shown in Table 2 along with the distribution guidelines implemented and the average items distributed in year 2 before guidelines were implemented. On average, students received more items when guidelines were implemented than when they were not.

**TABLE 2:** The average number of items distributed per visit per year when distribution guidelines were implemented and when they were not

	Y1 guidelines	Y1 distributed Mean (SD)	Y2 guidelines	Y2 distributed with guidelines Mean (SD)	Y2 distributed without guidelines Mean (SD)
Dairy	4	1.48 (1.10)	3	2.93 (1.37)	1.7 (1.26)
Protein	2	1.5 (1.16)	1	1.68 (0.96)	1.7 (0.57)
Produce	8	4.16 (3.10)	5	3.36 (1.89)	3.05 (1.47)
Fruit	4	2.04 (1.42)	—	1.88 (1.36)	1.7 (0.98)
Vegetables	4	2.11 (2.39)	—	1.76 (1.02)	1.35 (1.04)
Grain	2	2.31 (1.72)	2	2.88 (1.03)	1.85 (0.67)
Other items	4	5.29 (2.06)	11	6.27 (2.88)	4.95 (1.54)
Total items	20	14.53 (5.52)	22	17.41 (5.02)	13.35 (3.22)

The ability of students to follow the guidelines also depends on the number of items available per food category with dairy and produce being limited in some openings. On average, 1.35 fewer dairy items were distributed when adequate stock was not available (*P* = 0.000), equating to 6.5 fewer servings of dairy distributed (*P* = 0.089). When produce was not adequately stocked, students received on average 1.6 fewer produce items (*P* = 0.003), which equated to 3.3 fewer servings being received (*P* = 0.090). When there was not enough dairy or produce in stock, there were no significant changes in the number of other items distributed meaning students were not substituting other times in place of foods that were limited or not available.

Lastly, various adaptations were made during pantry implementation to keep up with the demand and growing reach of the pantry. Adaptations including the change in guidelines, the addition of 2 freezers, a second fridge, and more days spent restocking were outlined in the adoption section.

## Discussion

This study assessed the implementation and effectiveness of a client-choice campus food pantry to understand its potential impact on nutrition security and to inform future implementation. On average, the food distributed provided less than what is required for 3 d of intake for vitamin D, fruits, vegetables, and whole grains for all students. It also provided limited energy, fiber, potassium, vitamin A, and grains for male students, restricting the pantry’s ability to support nutrition security. The pantry provided not only more than what is required for 5 d of intake for most B vitamins, selenium, and grams of carbohydrates and protein but also sodium, added sugar, and saturated fat. Energy only met ∼3 d of intake underscoring the nutrient density of the food provided. As the pantry grew in use, it made adaptations to increase the number of restocking days and added more refrigerators and freezers to accommodate more food storage. However, during months of peak use, the pantry ran out of food to meet distribution guidelines for openings that averaged 34 client visits compared with 24. On days when there was not enough produce or dairy to meet distribution guidelines, the mean servings distributed per visit were reduced by 3.3 servings of produce and 6.5 servings of dairy. Taken together, these findings suggest that the ability of a pantry to support nutrition security may be subject to how the pantry is implemented (distribution guidelines and the type, quantity, and quality of food available). This is often driven by contextual factors like the pantry’s capacity for storage and restocking and the degree to which nutrition is embedded into the organizational culture.

The food groups received in limited amounts—fruits, vegetables, and whole grains—are consistent with those underconsumed by both college students [16] and the general United States population [29]. These food groups are essential to nutrition security as they prevent chronic disease and promote well-being due to their fiber and nutrient density [29]. Previous literature has also found that food insecurity puts college students at higher risk of inadequate nutrition, that is, consuming lower amounts of healthy foods (for example, fruits, vegetables, and whole grains) and more unhealthy foods (for example, fast foods, added sugars, and sugar-sweetened beverages) [16]. High amounts of sodium, added sugar, and saturated fat were also provided. Therefore, if food pantries are not able to provide students with adequate amounts of key food groups, or if they provide an excess of nutrients that should be limited, it is likely nutrition security will not be supported. Furthermore, campus food pantries that are understocked or do not support the distribution of key food groups will not be an effective intervention to close health disparity gaps that exist between food-secure and food-insecure students.

Students received limited amounts of vitamin D, which is a nutrient of concern for the general United States population [29], and was found to be provided in sparse amounts from another campus food pantry study [22]. Compared with research from traditional pantries (finding limited amounts of vitamins C, calcium, and dairy products were provided) [18] and a study from food pantries near a college campus (finding limited vitamin C and calcium were provided) [22], this campus client-choice pantry provided enough vitamin C, calcium, and dairy to meet at least 3 d of the recommended intake for both male and female students. In this study, there were more nutrients of concern for male students, given the fact that their daily intake requirements are higher than those for females and these nutrients of concern were consistent with previous literature: limited energy, potassium, and vitamin A [17,18,22]. Another study on food pantries near college campuses found low energy and high amounts of added sugar and trans-fat being distributed [22]. These findings are similar to our findings of higher amounts of added sugar and saturated fat relative to the amount of energy provided. Moving forward, these nutrients and food groups of concern need to be prioritized in both pantry stock and items distributed so that gaps in dietary disparities do not widen between those with consistent access to healthy food to those without.

Differences between the implementation of the pantry in year 1 compared with year 2 led to differences in items stocked and distributed. For example, there was more freezer and refrigerator space, different graduate students in charge of ordering and restocking, a change in campus programs donating food to the pantry, more restocking days directly from the food bank, and a change in distribution guidelines. One of the differences between year 1 and year 2 was more dairy and less produce to meet distribution guidelines. There was also an increase in the number of dairy, fruit, and vegetables distributed in year 2 but no significant change in grams or milliliters distributed. This is due to smaller-sized items of dairy, fruits, and vegetables being distributed in year 2 (for example, distributing cheese sticks compared with a block of cheese). The same goes for protein, with the same number of items being distributed, but larger-sized packages of protein were distributed, increasing the total grams of protein. There were also more items distributed, on average, in year 2 once limits were in place than there were when no guidelines were in effect. This is particularly interesting given that the limits were initially implemented to address concerns that the pantry was running out of food.

While the implementation of distribution guidelines did lead to more items distributed, it also led to more nutrients and servings of food distributed with foods coming from all food groups. Implementing distribution guidelines may be one way to preserve client choice and respect the client’s ability to select culturally appropriate foods, while also supporting dietary recommendations. The ability of clients to select items that correspond with guidelines, however, depends on there being adequate stock for major food categories. It is also important to note, that a campus food pantry’s potential to promote nutrition security may come down to the capacity of the pantry to provide enough healthy foods for the number of student visitors. Previous research has found that campus pantries grow in the number of students served over time [12,30], and therefore, it is likely that campus pantries will need to adapt their program implementation to keep up with demand or further limit the number of items students can take to ensure that there is enough food for all students seeking support. Food pantries, and particularly campus food pantries, have limitations like food storage (refrigerated space and shelf space) and the capacity to restock (for example, limited time, workforce, or money to purchase more food). These limitations were mentioned by staff regarding the amount of food they could transport to and from the food bank and the storage limitations in the space they had available. Therefore, implementing distribution guidelines that allow students to take many items, or items from all food groups may not be feasible. Furthermore, it may not be realistic for food pantries to be the only food assistance resource on college campuses nor a reasonable resource for students in need of long-term or substantial assistance.

This study added to the sparse literature on the food and nutrients received from client-choice campus pantries; however, there were limitations. There was a reduction in sample size due to missing data (3.2% of pantry openings were excluded) and multiple imputation was not feasible due to the limited amount of data variation and missingness within the sample. Influences in the supply chain or other logistical limitations were not assessed to determine potential causes that led to inadequate stock. To the best of our knowledge, there were no known budget constraints in either year of operation. Pantry staff mentioned efforts to try to restock pantry items during openings when food was running out, but this was not assessed. We were also not able to assess whether having graduate students from dietetic backgrounds was particularly helpful for pantry operations or the distribution of healthy foods. Lastly, maintenance was not assessed in the present study and should be included in future evaluations.

Implementing distribution guidelines at a client-choice campus food pantry may support nutrition security while honoring food preferences. Distribution guidelines may be one way to bridge the research-to-practice gap if guidelines implemented follow evidence-based dietary recommendations like MyPlate. Campus food pantries should consider increasing their food storage capacity, especially for refrigerated or freezer storage, when possible, to support the adequate stock of dairy, produce, and perishable protein sources. Food pantries should consider placing a greater emphasis on stocking, distributing, and/or providing nutrition education on foods rich or fortified with vitamin D, given it was the only nutrient provided in limited amounts for both males and females and is a nutrient of concern for the general United States population. Male students may need access to more food to meet sex-specific DRIs. The use of a nutritional ranking system is another option to move the charitable food system from supporting food sufficiency to promoting nutrition security, as recommended by the AHA [8]. A nutritional ranking system could be used in conjunction with distribution guidelines, given not all items within the same food group are necessarily equal in the nutritional value they provide (for example, full-fat dairy compared with low-fat dairy, whole-grain pasta compared with regular pasta, etc.). Flagging items by nutritional rank using a system such as the Healthy Eating Research Nutrition Guidelines for the Charitable Food System could be one way to promote healthier food selections (that is, foods with low amounts of added sugar, sodium, and saturated fat) from each food group. This nutritional ranking system uses a stoplight color approach to quickly identify foods based on their nutritional value (green “choose often,” yellow “choose sometimes,” and red “choose rarely”). This color system could also be useful for pantry staff to quickly identify which foods need to be restocked or inform the organization of food on pantry shelves if choice architecture is used to promote healthy food selections. If the pantry were to recreate distribution guidelines based on evidence-based practices like MyPlate, use a nutritional ranking system, or choice architecture, having graduate students in dietetics may be helpful as opposed to students from other academic backgrounds.

More research is needed to examine ways to move food-insecure students from food sufficiency to nutrition security. Particularly more research is needed to determine the food and nutrients distributed under different implementation methods, such as using distribution guidelines or a nutritional ranking system. In future campus food pantry research, it will be important to document pantry implementation to make findings more generalizable. Future studies analyzing campus food pantries should identify how specific implementation strategies impact program reach, effectiveness, adoption, and maintenance. The creation of a survey or other tool to measure nutrition security among the college student population is also needed. Future research should seek to understand how food distributed from campus pantries contributes to students’ overall dietary quality. Colleges and universities should consider other ways to promote nutrition security through the implementation of policy and systems changes that would help students facing persistent food insecurity. Ideas include more affordable meal plans for students based on financial need, like what is offered by the National School Lunch Program (that is, free, or reduced-price meals), or implementing policies around free food events to prioritize or incentivize the use of healthier options. Free cooking and food resource management classes could also help improve student food literacy and promote life-long, food resource management habits.

## Funding

The Food Assistance & Well-being Program at the University of Illinois at Urbana-Champaign aided in data collection. Funding for AIM came from the National Science Foundation Graduate Research Fellowship Program.

## Author contributions

The authors’ responsibilities were as follows – AIM, MPP: designed research; AIM: analyzed data; AIM: wrote the paper; and both authors: edited and approved the final manuscript.

## Conflicts of interest

The authors report no conflicts of interest.

## Data availability

Data described in the manuscript, code book, and analytic code will not be made available because data do not belong to the authors. Requests for data access can be sent to the corresponding author, and she will forward the request to the appropriate parties.
